# Supplementary material for: Screening an In-House Isoquinoline Alkaloids Library for New Blockers of Voltage-Gated Na+ Channels Using Voltage Sensor Fluorescent Probes: Hits and Biases
Source: Molecules. 2022 Jun 28;27(13):4133. doi: 10.3390/molecules27134133 (PMC9268414; doi:10.3390/molecules27134133)
Supplement: Supplementary file 1 [file molecules-27-04133-s001.zip › Figure S1 RMN oxostephanine.pdf]

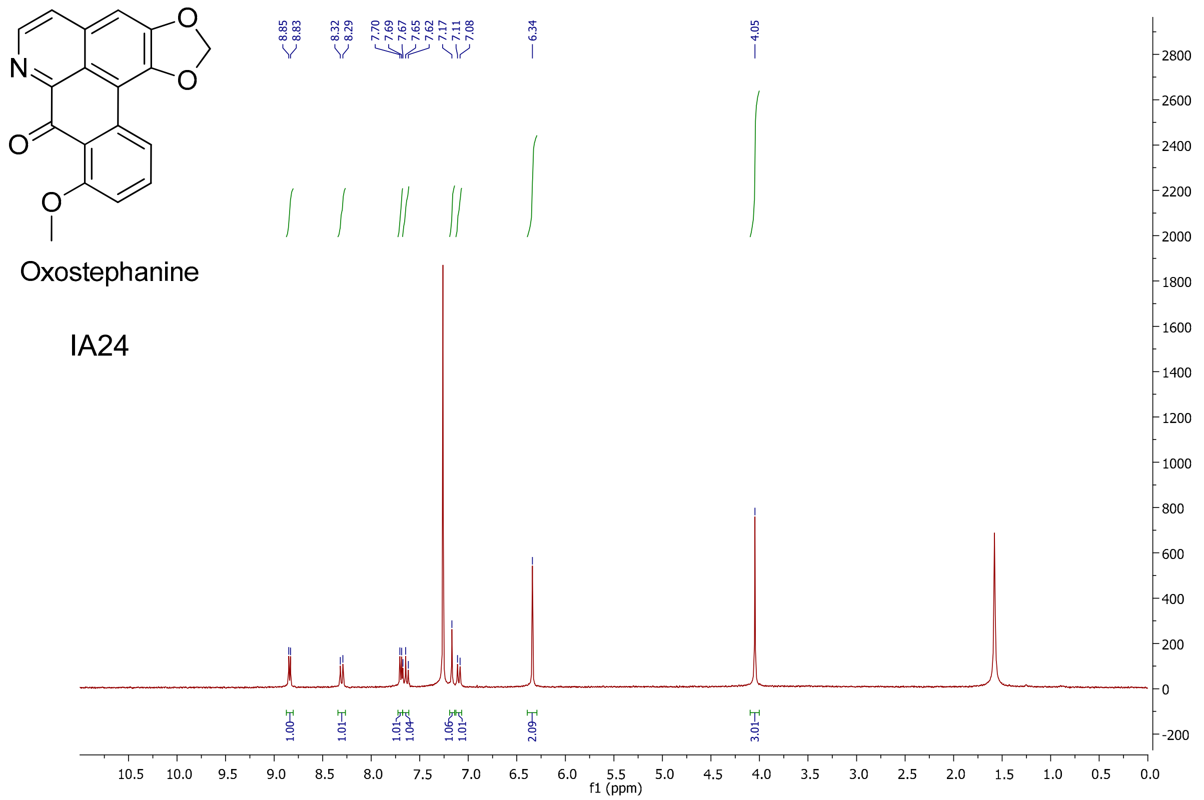

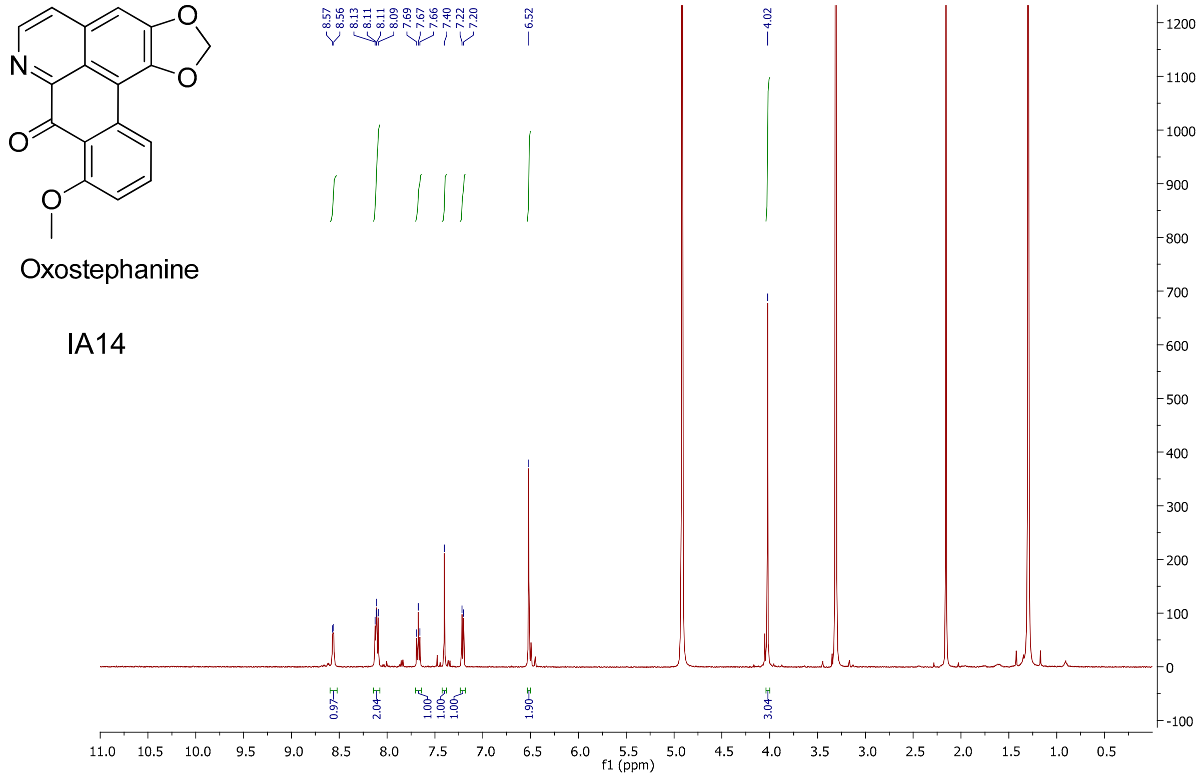

Supplementary Figure S1. ^{1}H NMR spectrum of IA24 and IA14 (oxostephanine) recorded in $CDCl_3$ at 300 MHz.
