# Supplementary material for: Screening an In-House Isoquinoline Alkaloids Library for New Blockers of Voltage-Gated Na+ Channels Using Voltage Sensor Fluorescent Probes: Hits and Biases
Source: Molecules. 2022 Jun 28;27(13):4133. doi: 10.3390/molecules27134133 (PMC9268414; doi:10.3390/molecules27134133)
Supplement: Supplementary file 1 [file molecules-27-04133-s001.zip › Figure S6.pdf]

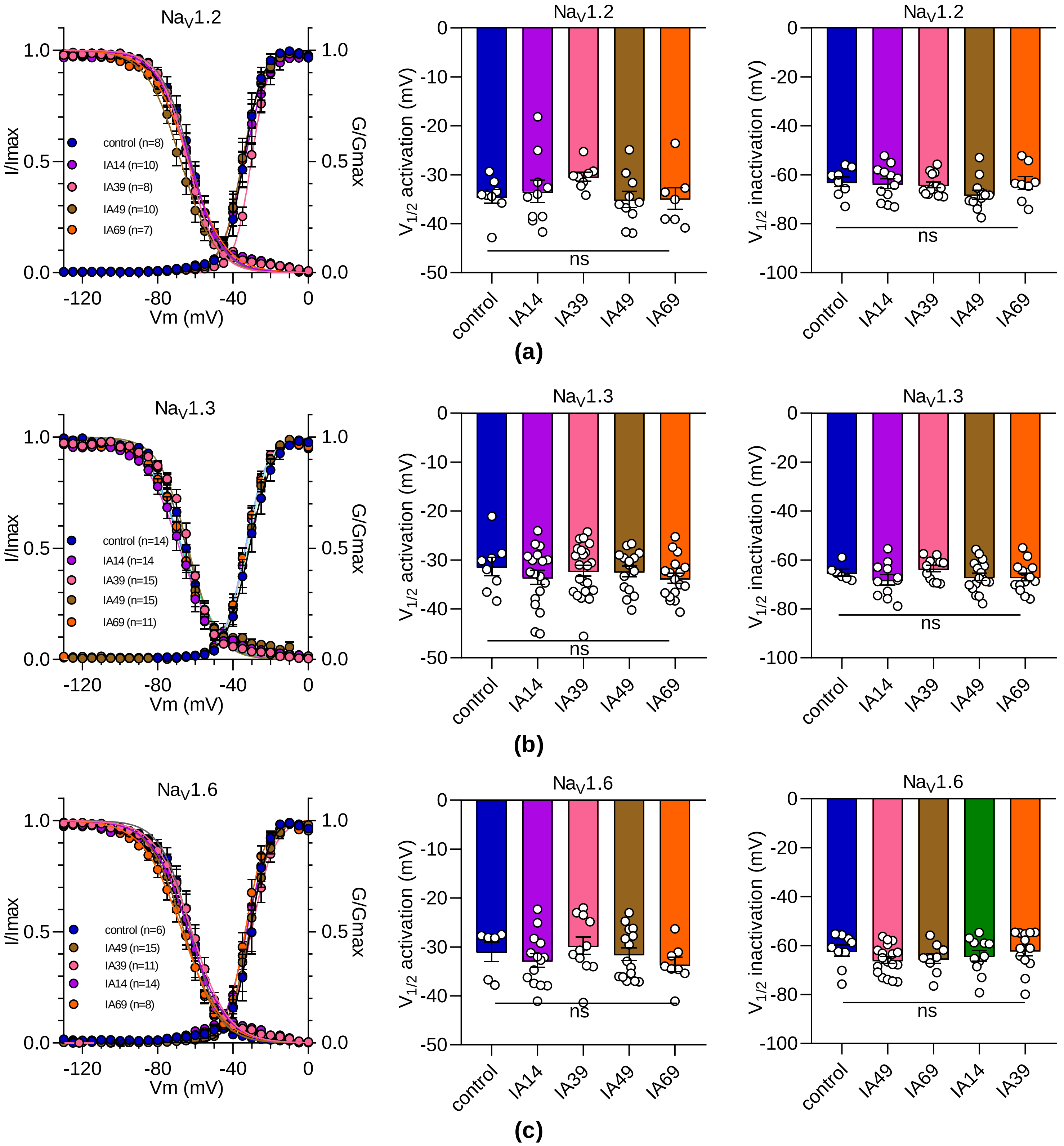

Supplementary Figure S6. Effects of oxostephanine, liriodenine, thalmiculine, and protopine on activation and inactivation properties of hNa_V1.2, hNa_V1.3 and hNa_V1.6 channels.

Oxostephanine, liriodenine, thalmiculine, and protopine were assayed at 10 μM each, on Na^+ currents elicited by hNa_V1.2 **(a)**, hNa_V1.3 **(b)** and hNa_V1.6 **(c)** channels stably expressed in HEK293T cells. From the left to the right: activation and inactivation curves of I_{Na} recorded in the absence (control, DMSO 0.3% in extracellular solution) and in the presence of oxostephanine (IA14), liriodenine (IA39), thalmiculine (IA49), and protopine (IA69). The histograms illustrate the comparison of $V_{1/2}$ activation and inactivation in the absence (control) and in the presence of oxostephanine (IA14), liriodenine (IA39), thalmiculine (IA49), and protopine (IA69), respectively. Data are mean±SEM. Statistical analysis were performed using one-way ANOVA test followed by Dunnett's multiple comparison tests. n.s: not significant.
