# Supplementary material for: Screening an In-House Isoquinoline Alkaloids Library for New Blockers of Voltage-Gated Na+ Channels Using Voltage Sensor Fluorescent Probes: Hits and Biases
Source: Molecules. 2022 Jun 28;27(13):4133. doi: 10.3390/molecules27134133 (PMC9268414; doi:10.3390/molecules27134133)
Supplement: Supplementary file 1 [file molecules-27-04133-s001.zip › Figuse S7-2022.pdf]

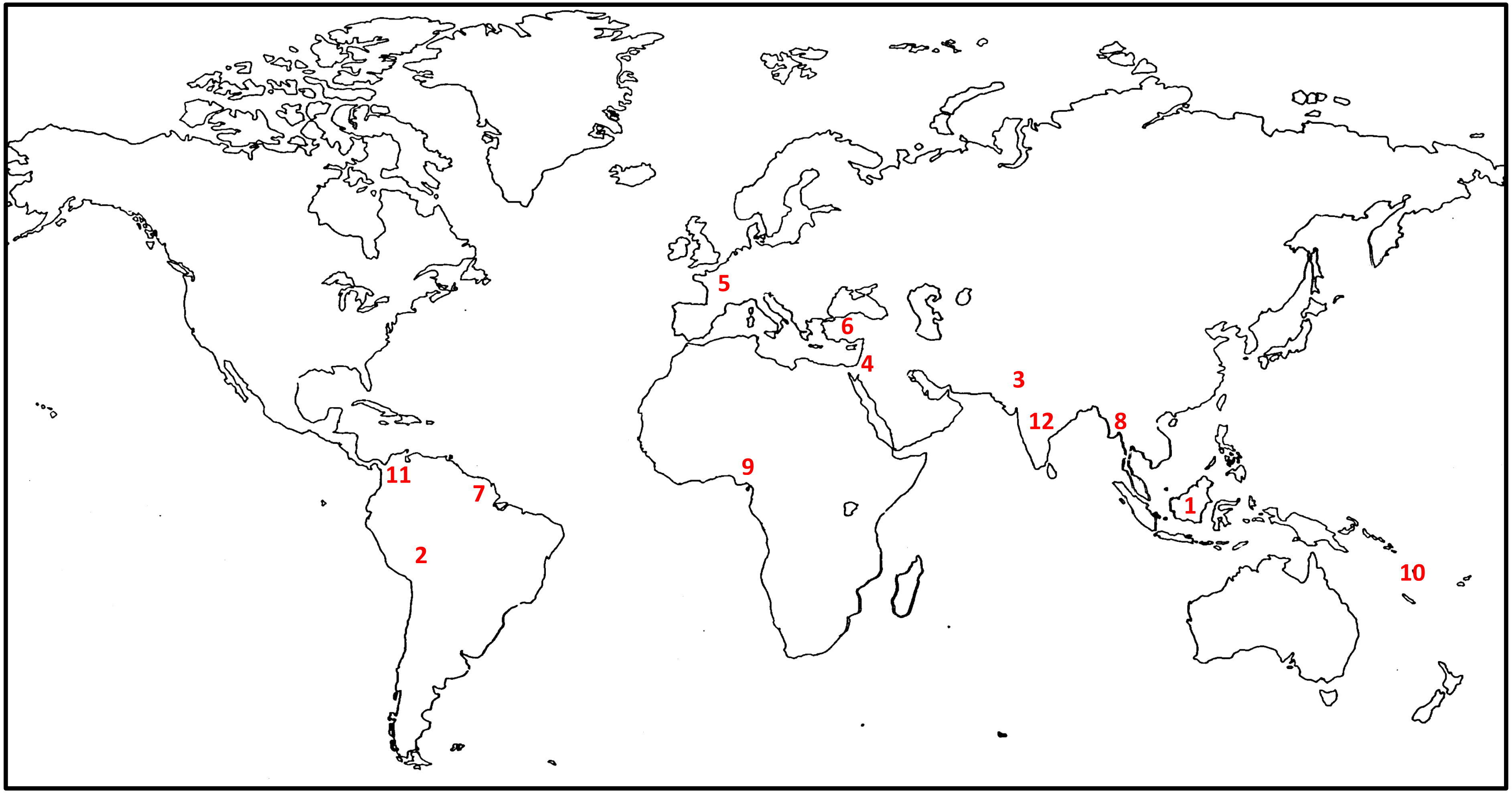

Figure S7. World map showing the geographical origins of plants used to purify the 62 isoquinoline alkaloids screened for voltage-gated Na^+ channel blockers.

1. Indonesia (*Albertisia papuana, Menispermaceae*)
2. Bolivia (*Aniba canelilla* H.B.K., *Lauraceae*)
3. Pakistan (*Berberis lycium, Berberidaceae* ; *Thalictrum cultratum, Ranunculaceae*)
4. Jordan (*Ceratocapnos palaestinus, Fumariaceae*)
5. France (*Corydalis claviculata, Papaveraceae*)
6. Turkey (*Corydalis majori, Fumariaceae* ; *Thalictrum minus* var. *microphyllum, Ranunculaceae*)
7. French Guyana (*Curarea candicans, Menispermaceae*)
8. Thailand (*Cyclea atjehensis, Menispermaceae* ; *Cyclea barbata, Menispermaceae* ; *Pachygone dasycarpa, Menispermaceae* ; *Stephania pierrii (synonym Stephania erecta), Menispermaceae* ; *Stephania suberosa, Menispermaceae* ; *Stephania venosa, Menispermaceae*)
9. Nigeria (*Glossocalyx brevipes, Siparunaceae*)
10. Vanuatu (*Gyrocarpus americanus, Hernandiaceae* ; *Pycnarrhena sp., Menispermaceae*)
11. Colombia (*Pseudoxandra lucida, Annonaceae*)
12. India (*Tiliacora racemosa, Menispermaceae*)
