# Supplementary material for: Screening an In-House Isoquinoline Alkaloids Library for New Blockers of Voltage-Gated Na+ Channels Using Voltage Sensor Fluorescent Probes: Hits and Biases
Source: Molecules. 2022 Jun 28;27(13):4133. doi: 10.3390/molecules27134133 (PMC9268414; doi:10.3390/molecules27134133)
Supplement: Supplementary file 1 [file molecules-27-04133-s001.zip › Table S1-2022.pdf]

Supplementary Table S1. Alkaloids used in this work to screen for Na_v channels inhibitors.

Sample number	Trivial name	Chemical class (CAS number)	Structure	Plant origin (part of plant)	Reference
IA1	(-)-Asimilobine	Aporphine (6871-21-2)	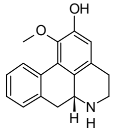	*Glossocalyx brevipes, Siparunaceae* (whole plant)	Montgomery C.T., 1985
IA2	(-)-Argemonine	Pavine (6901-16-2)	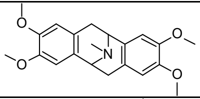	*Cyclea atjehensis, Menispermaceae* (whole plant)	Tantisewie B., 1989
IA3	(-)-Norargemonine (1st batch)	Pavine (5876-16-4)	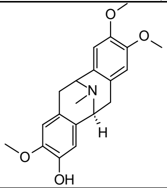	*Cyclea atjehensis, Menispermaceae* (whole plant)	Tantisewie B., 1989 (1)
IA4	(-)-Cycleanine	BBIQ (518-94-5)	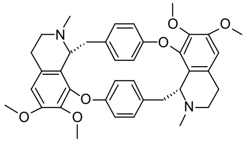	*Stephania pierrii (synonym Stephania erecta), Menispermaceae* (tuberous roots)	Tantisewie B., 1989 (2)
IA5	(-)-2-Northalmine	BBIQ (101488-79-3)	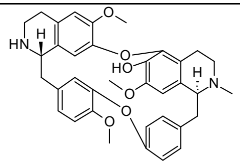	*Thalictrum cultratum, Ranunculaceae* (whole plant)	Hussain S.F., 1985
IA6	(+)-Cepharanthine (1st batch)	BBIQ (481-49-2)	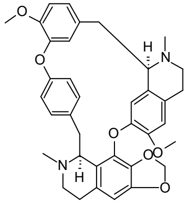	*Stephania suberosa, Menispermaceae* (tuberous roots)	Patra A., 1986

IA7	(+)-Cepharanthine (2nd batch)	BBIQ (481-49-2)	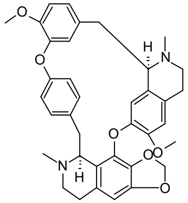	*Stephania suberosa, Menispermaceae* (tuberous roots)	Patra A., 1986
IA8	(+)-Cepharanthine (3rd batch)	BBIQ (481-49-2)	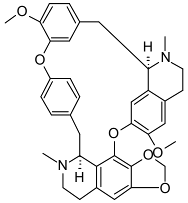	*Stephania suberosa, Menispermaceae* (tuberous roots)	Patra A., 1986
IA9	(-)-Thalrugosaminine	BBIQ (22226-73-9)	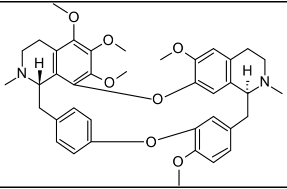	*Thalictrum cultratum, Ranunculaceae* (whole plant)	Hussain S.F., 1986
IA10	Tetrahydropalmatine	Protoberberine (2934-97-6)	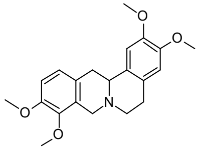	*Berberis sp., Berberidaceae* (unknown)	Unpublished results
IA11	(-)-Kikemanine (1st batch)	Protoberberine (30413-84-4)	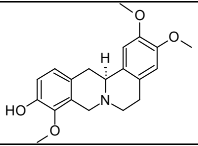	*Stephania venosa, Menispermaceae* (leaves)	Pharadai K., 1985
IA13	Protopine (1st batch)	Protopine (130-86-9)	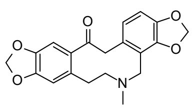	*Corydalis majori, Fumariaceae* (whole plant)	Allais D.P., 1988
IA14	Oxostephanine (1st batch)	Oxoaporphine (58262-58-1)	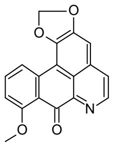	*Stephania venosa, Menispermaceae* (leaves)	Pharadai K., 1985

IA15	(-)-Limacine	BBIQ (10172-02-8)	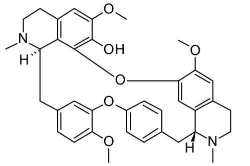	*Curarea candicans, Menispermaceae* (roots)	Lavault M., 1985
IA17	(-)-Curicycleatjenine	Amidic BBIQ (131984-83-3)	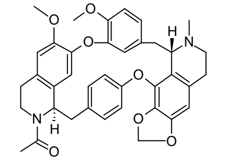	*Cyclea atjehensis, Menispermaceae* (whole plant)	Tantisewie B., 1989
IA19	(-)-Isocuricycleatjine	Amidic BBIQ (131984-86-6)	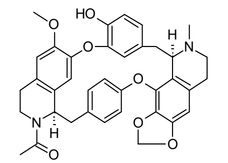	*Cyclea atjehensis, Menispermaceae* (leaves)	Tantisewie B., 1990 (1, 2)
IA20	(-)-Pseudoanibacanine	Protoberberine (151851-39-7)	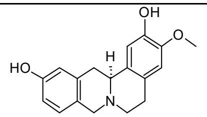	*Aniba canelilla* H.B.K.*, Lauraceae* (stem barks)	Oger J.M., 1992
IA21	(-)-(8α)-Methyl-Pseudo anibacanine	Protoberberine (151851-40-0)	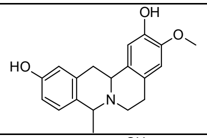	*Aniba canelilla* H.B.K.*, Lauraceae* (stem barks)	Oger J.M., 1992
IA22	(-)-Anibacanine	Protoberberine (151757-06-1)	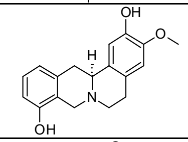	*Aniba canelilla* H.B.K.*, Lauraceae* (stem barks)	Oger J.M., 1992
IA24	Oxostephanine (2nd batch)	Oxoaporphine (58262-58-1)	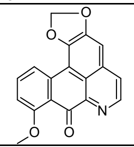	*Stephania venosa, Menispermaceae* (leaves)	Pharadai K., 1985

IA26	(+)-Limacusine	BBIQ (10172-03-9)	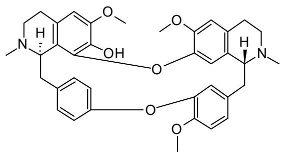	*Curarea candicans, Menispermaceae* (roots)	Lavault M., 1985
IA27	Fraction containing : (+)-Berbamine and Oxyacanthine	BBIQ (478-61-5) + BBIQ (548-40-3)	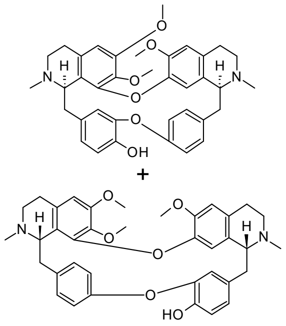	*Berberis lycium, Berberidaceae* (unknown)	Hussain S.F. Unpublished results
IA28	(+)-Daphnoline	BBIQ (479-36-7)	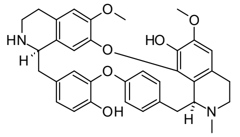	*Albertisia papuana, Menispermaceae* (liana)	Lavault M., 1987
IA30	(+)-Laurotetanine	Aporphine (128-76-7)	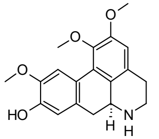	*Glossocalyx brevipes, Siparunaceae* (whole plant)	Montgomery C.T., 1985
IA31	Tiliacorinine	BBIQ (27073-72-9)	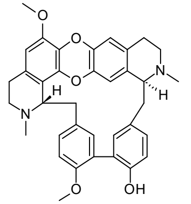	*Tiliacora racemosa, Menispermaceae* (unknown)	Shamma M., 1976

IA32	Thalmelatidine	Aporphine-BIQ (31199-55-0)	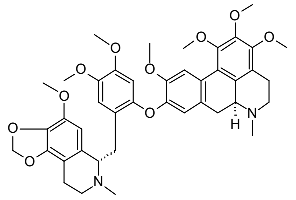	*Thalictrum sp., Ranunculaceae* (unknown)	Guinaudeau H. Unpublished results
IA33	Antioquine (S,R) (1st batch)	BBIQ (93767-27-2)	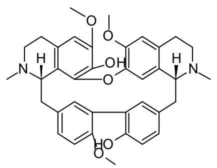	*Pseudoxandra lucida, Annonaceae* (stem bark)	Cortes D., 1985
IA34	(-)-Kikemanine (2nd batch)	Protoberberine (30413-84-4)	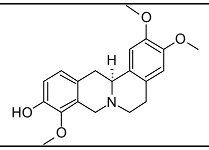	*Stephania venosa, Menispermaceae* (rhizomes)	Charles B., 1987
IA35	Pallidine	Morphinandione (25650-75-3)	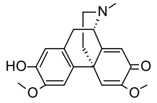	*Pycnarrhena sp., Menispermaceae* (unknown)	Pharadai K. Unpublished results
IA36	Sukhodianine	Aporphine (82413-17-0)	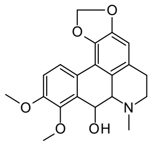	*Stephania venosa, Menispermaceae* (leaves)	Pharadai K., 1985
IA39	Liriodenine (2nd batch)	Oxoaporphine (475-75-2)	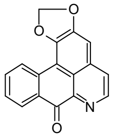	*Stephania venosa, Menispermaceae* (leaves)	Pharadai K., 1985
IA40	Thaliphylline	BBIQ (93780-79-1)	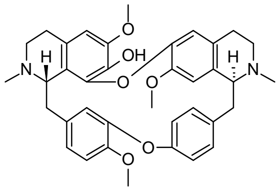	*Thalictrum minus* var. *microphyllum, Ranunculaceae* (roots and rhizomes)	Guinaudeau H., 1984

IA41	(-)-Curine	BBIQ (436-05-5)	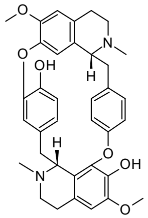	*Cyclea barbata, Menispermaceae* (roots)	Guinaudeau H., 1993
IA42	Gyrocarpusine	BBIQ (102518-66-1)	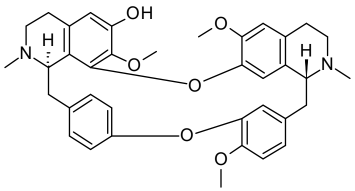	*Gyrocarpus americanus, Hernandiaceae* (stem bark)	Chalandre M.C., 1986
IA44	(+)-Homoaromoline	BBIQ (17132-74-0)	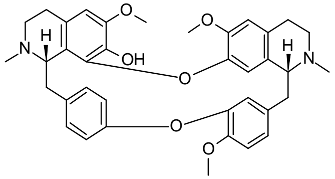	*Gyrocarpus americanus, Hernandiaceae* (stem bark)	Chalandre M.C. Unpublished results
IA45	(+/-)-8-oxotetrahydropalmatine	Protoberberine (81701-50-0)	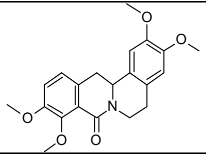	*Pycnarrhena sp., Menispermaceae* (unknown)	Pharadai K. Unpublished results
IA46	(+)-Aromoline	BBIQ (519-53-9)	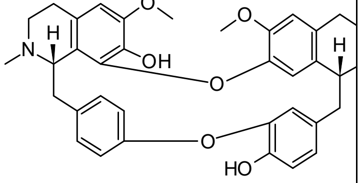	*Albertisia papuana, Menispermaceae* (stems)	LeBoeuf M., 1982
IA47	(+)-Isoboldine	Aporphine (3019-51-0)	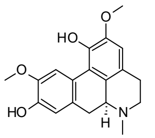	*Aniba canelilla* H.B.K.*, Lauraceae* (stem barks)	Oger J.M., 1992

IA48	(+)-Claviculine	Cularine (87035-67-4)	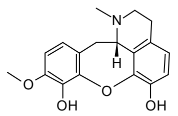	*Corydalis claviculata, Papaveraceae* (whole plant)	Allais D.P., 1990
IA49	(-)-Thalmiculine	BBIQ (106146-69-4)	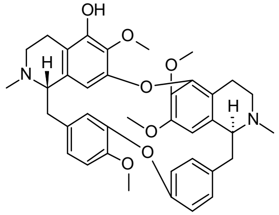	*Thalictrum cultratum, Ranunculaceae* (whole plant)	Hussain S.F., 1986
IA50	(+)-Tetrandrine	BBIQ (518-34-3)	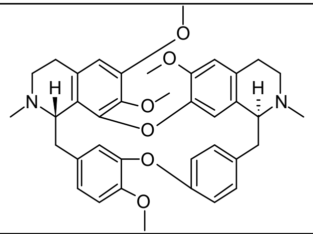	*Pachygone dasycarpa, Menispermaceae* (stem bark)	Guinaudeau H., 1997
IA51	Isochondodendrin e	BBIQ (477-62-3)	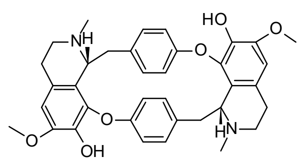	*Curarea candicans, Menispermaceae* (roots)	Lavault M., 1985
IA52	(+)-Bebeerine	BBIQ (477-60-1)	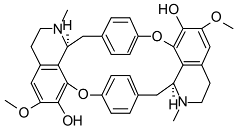	*Curarea candicans, Menispermaceae* (roots)	Lavault M., 1985
IA53	Oxosarcocapnidin e	Cularine (87035-68-5)	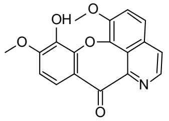	*Ceratocapnos palaestinus, Fumariaceae* (whole plant)	Herath W.H.M.W, 1990

IA54	(+)-Celtine	Cularine (91106-26-2)	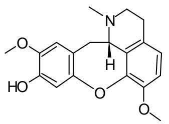	*Ceratocapnos palaestinus, Fumariaceae* (whole plant)	Herath W.H.M.W, 1990
IA62	(-)-Norargemonine (2nd batch)	Pavine (5876-16-4)	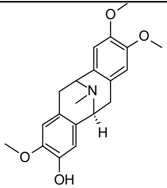	*Cyclea atjehensis, Menispermaceae* (whole plant)	Tantisewie B., 1989
IA69	Protopine (2nd batch)	Protopine (130-86-9)	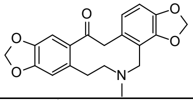	*Corydalis majori, Fumariaceae* (whole plant)	Allais D.P., 1988
IA73	Antioquine (S,R) (2nd batch)	BBIQ (93767-27-2)	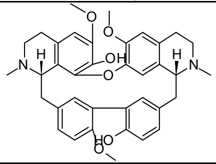	*Pseudoxandra lucida, Annonaceae* (stem bark)	Cortes D., 1985
IA76	(S)-Stylopine (1st batch)	Berberine (84-39-9)	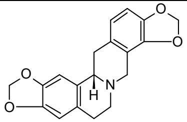	*Corydalis majori, Fumariaceae* (whole plant)	Allais D.P., 1988
IA77	(S)-Stylopine (2nd batch)	Berberine (84-39-9)	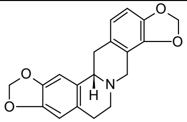	*Corydalis majori, Fumariaceae* (whole plant)	Allais D.P., 1988

*Fractions are represented by IA55, 61, 64-65, 67-68, 70-72, 74 and 80-81

IA55 (*Ceratocapnos palaestinus*, Herath W.H.M.W, 1990),
61 (*Stephania pierrii,* Tantisewie B., 1989*)*,
64 (*Pachygone dasycarpa,* Guinaudeau H., 1997)
65 (*Tiliacora warneckei,* Unpublished results)
67 (*Albertisia papuana,* Abouchacra M.L., 1982)
68 (*Corydalis majori*, Allais D.P., 1988)
70 (*Corydalis majori*, Allais D.P., 1988)
71 (*Gyrocarpus americanus*, unpublished results)
72 (*Pseudoxandra* sp, unpublished results)
74 (*Pachygone dasycarpa,* Guinaudeau H., 1997)
80 (*Pachygone dasycarpa,* Guinaudeau H., 1997)
81 (*Pachygone dasycarpa,* Guinaudeau H., 1997)

References

Allais, D.-P.; Gozler, T.; Guinaudeau, H., Alkaloids from *Corydalis majori* Poelln. (*Corydalis integra* Barbey et Major) (Fumariaceae). *Plant. Med. Phytother.* **1988,** *22* (4), 219-24.

Allais, D.-P.; Guinaudeau, H., Composition alcaloïdique de *Corydalis claviculata*. *J. Nat. Prod.* **1990,** *53* (5), 1280-6. DOI : 10.1021/np50071a022

Chalandre, M.-C.; Bruneton, J.; Cabalion, P.; Guinaudeau, H., Alcaloïdes de *Gyrocarpus americanus*. *J. Nat. Prod.* **1986,** *49* (1), 101-105. DOI: 10.1021/np50043a011

Charles, B.; Bruneton, J.; Pharadai, K.; Tantisewie, B.; Guinaudeau, H.; Shamma, M., Some unusual proaporphine and aporphine alkaloids from *Stephania venosa*. *J. Nat. prod.* 1987, 50(6), 1113-17. DOI : 10.1021/np50054a017

Cortes, D.; Saez, J.; Hocquemiller, R.; Cave, A.; Cave, A., Alcaloïdes des Annonacées. LIII. Alcaloïdes du *Pseudoxandra aff. lucida*. Etude de l'antioquine et de ses dérivés. *J. Nat. Prod.* **1985,** *48* (1), 76-85. DOI : 10.1021/np50037a014

Guinaudeau, H.; Freyer, A. J.; Shamma, M.; Husnu, K.; Baser, C., Enzymic control of stereochemistry among the *Thalictrum* bisbenzylisoquinoline alkaloids. *Tetrahedron* **1984,** *40* (11), 1975-82. DOI : 10.1016/S0040-4020(01)88437-4

Guinaudeau, H.; Lin, L. Z.; Ruangrungsi, N.; Cordell, G. A., Bisbenzylisoquinoline alkaloids from *Cyclea barbata*. *J. Nat. Prod.* **1993,** *56* (11), 1989-92. DOI : 10.1021/np50101a020

Guinaudeau, H.; Böhlke, M.; Lin, L.-Z.; Angerhofer, C. K.; Cordell, G. A.; Ruangrungsi, N., (+)-Angchibangkine, a new type of bisbenzylisoquinoline alkaloid, and other dimers from *Pachygone dasycarpa*. *J. Nat. Prod.* **1997,** *60* (3), 258-260. DOI : 10.1021/np960568e

Herath, W. H. M. W.; Abu Zarga, M. H.; Sabri, S. S.; Guinaudeau, H.; Shamma, M., Some C-secocularines from *Ceratocapnos palaestinus*. *J. Nat. Prod.* **1990,** *53* (4), 1006-8. DOI : 10.1021/np50070a040

Hussain, S. F.; Guinaudeau, H.; Freyer, A.; Shamma, M., Bisbenzylisoquinoline alkaloids from *Thalictrum cultratum*. The structures of thalrugosinone and thalpindione. *J. Nat. Prod.* **1985,** *48* (6), 962-6. DOI : 10.1021/np50042a014

Hussain, S. F.; Freyer, A. J.; Guinaudeau, H.; Shamma, M., Five new bisbenzylisoquinoline alkaloids from *Thalictrum cultratum*. *J. Nat. Prod.* **1986,** *49* (3), 488-93. DOI : 10.1021/np50045a018

Lavault, M.; Fournet, A.; Guinaudeau, H.; Bruneton, J., Bisbenzylisoquinoline N-oxides from *Curarea candicans*. *J. Chem. Res., Synop.* **1985,** (8), 248-9.

Lavault, M.; Bruneton, J.; Cave, A.; Chan, K. C.; Deverre, J. R.; Sevenet, T.; Guinaudeau, H., Alcaloïdes bisbenzylisoquinolines de *Albertisia* cf. *A. papuana*. *Can. J. Chem.* **1987,** *65* (2), 343-7. DOI : 10.1139/v87-058

LeBoeuf, M.; Abouchacra, M. L.; Sevenet, T.; Cavé, A., Alcaloïdes de *Albertisia papuana* Becc., Menispermaceae. *Plant. Med. Phytother.* **1982,** *16* (4), 280-91.

Montgomery, C. T.; Freyer, A. J.; Guinaudeau, H.; Shamma, M.; Fagbule, M. O.; Olatunji, G., (+)-N-Methyllaurotetanine-β-N-oxide from *Glossocalyx brevipes*. *J. Nat. Prod.* **1985,** *48* (5), 833-4. DOI : 10.1021/np50041a025

Oger, J. M.; Duval, O.; Richomme, P.; Bruneton, J.; Guinaudeau, H.; Fournet, A., (R)-(+)-Noranicanine a new type of trioxygenated benzylisoquinoline isolation and synthesis. *Heterocycles* **1992,** *34* (1), 17-20. DOI : 10.3987/com-91-5903

Patra, A.; Freyer, A. J.; Guinaudeau, H.; Shamma, M.; Tantisewie, B.; Pharadai, K., The bisbenzylisoquinoline alkaloids of Stephania suberosa. *J. Nat. Prod.* **1986,** *49* (3), 424-7. DOI : 10.1021/np50045a006

Pharadai, K.; Pharadai, T.; Tantisewie, B.; Guinaudeau, H.; Freyer, A. J.; Shamma, M., (-)-O-Acetylsukhodianine and oxostephanosine: two new aporphinoids from *Stephania venosa*. *J. Nat. Prod.* **1985,** *48* (4), 658-9. DOI : 10.1021/np50040a028

Shamma, M.; Foy, J. E.; Govindachari, T. R.; Viswanathan, N., The position of the phenolic function in tiliacorine and related alkaloids. *J. Org. Chem.* **1976,** *41* (7), 1293-4. DOI : 10.1021/jo00869a059

Tantisewie, B.; Pharadai, T.; Pandhuganont, M.; Guinaudeau, H.; Freyer, A. J.; Shamma, M., (+)-N-Formylnornantenine, a new aporphine alkaloid from *Cyclea atjehensis*. *J. Nat. Prod.* **1989,** *52* (3), 652-4. DOI : 10.1021/np50063a032

Tantisewie, B.; Amurrio, S.; Guinaudeau, H.; Shamma, M., New bisbenzylisoquinolines from Stephania pierrii. *J. Nat. Prod.* **1989,** *52* (4), 846-51. DOI : 10.1021/np50064a031

Tantisewie, B.; Pharadai, K.; Amnauypol, S.; Freyer, A. J.; Guinaudeau, H.; Shamma, M., A new subgroup of bisbenzylisoquinoline alkaloids: (+)-cycleatjehenine and (+)-cycleatjehine. *Tetrahedron* **1990,** *46* (2), 325-30. DOI : 10.1016/S0040-4020(01)85416-8

Tantisewie, B.; Pharadai, T.; Freyer, A. J.; Guinaudeau, H.; Shamma, M., Four amidic bisbenzylisoquinoline alkaloids from *Cyclea atjehensis*. *J. Nat. Prod.* **1990,** *53* (3), 553-8. DOI : 10.1021/np50069a003
