# Supplementary figures and images for: Screening an In-House Isoquinoline Alkaloids Library for New Blockers of Voltage-Gated Na+ Channels Using Voltage Sensor Fluorescent Probes: Hits and Biases
Source: Molecules. 2022 Jun 28;27(13):4133. doi: 10.3390/molecules27134133 (PMC9268414; doi:10.3390/molecules27134133)

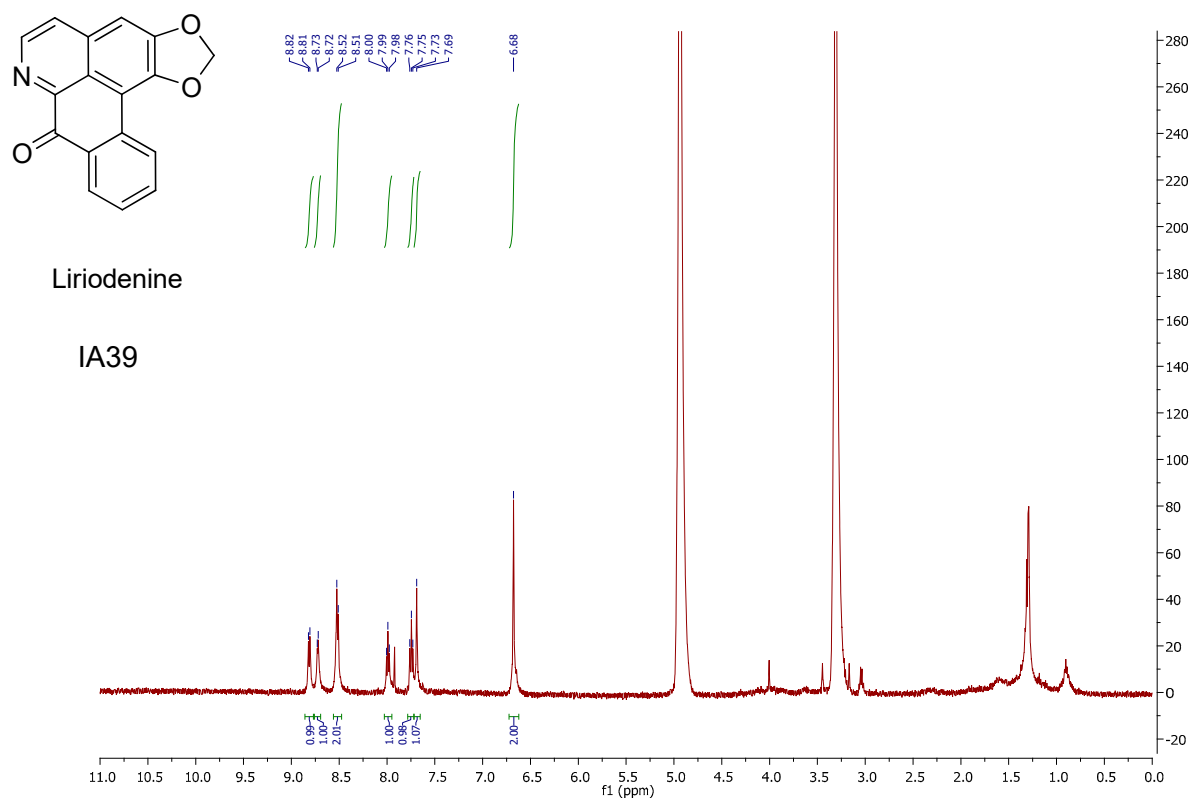

Figure S2. <sup>1</sup>H NMR spectrum of IA39 (liriodenine).

Supplement: Supplementary file 1 [file molecules-27-04133-s001.zip › Figure S2 RMN-liriodenine.pdf]

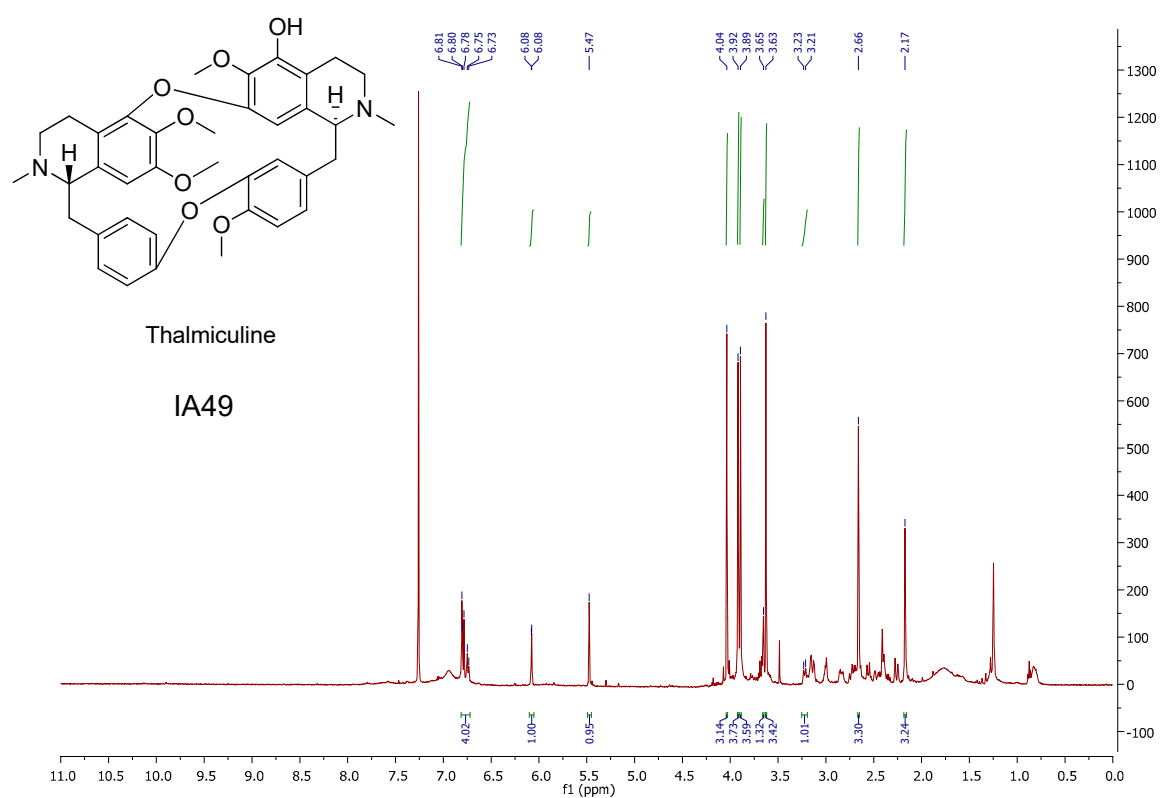

**Supplementary Figure S3.**  $^1\text{H}$  NMR spectrum of IA49 (thalmiculine) recorded in  $\text{CDCl}_3$  at 300 MHz.

Supplement: Supplementary file 1 [file molecules-27-04133-s001.zip › Figure S3-RMN thalmiculine.pdf]

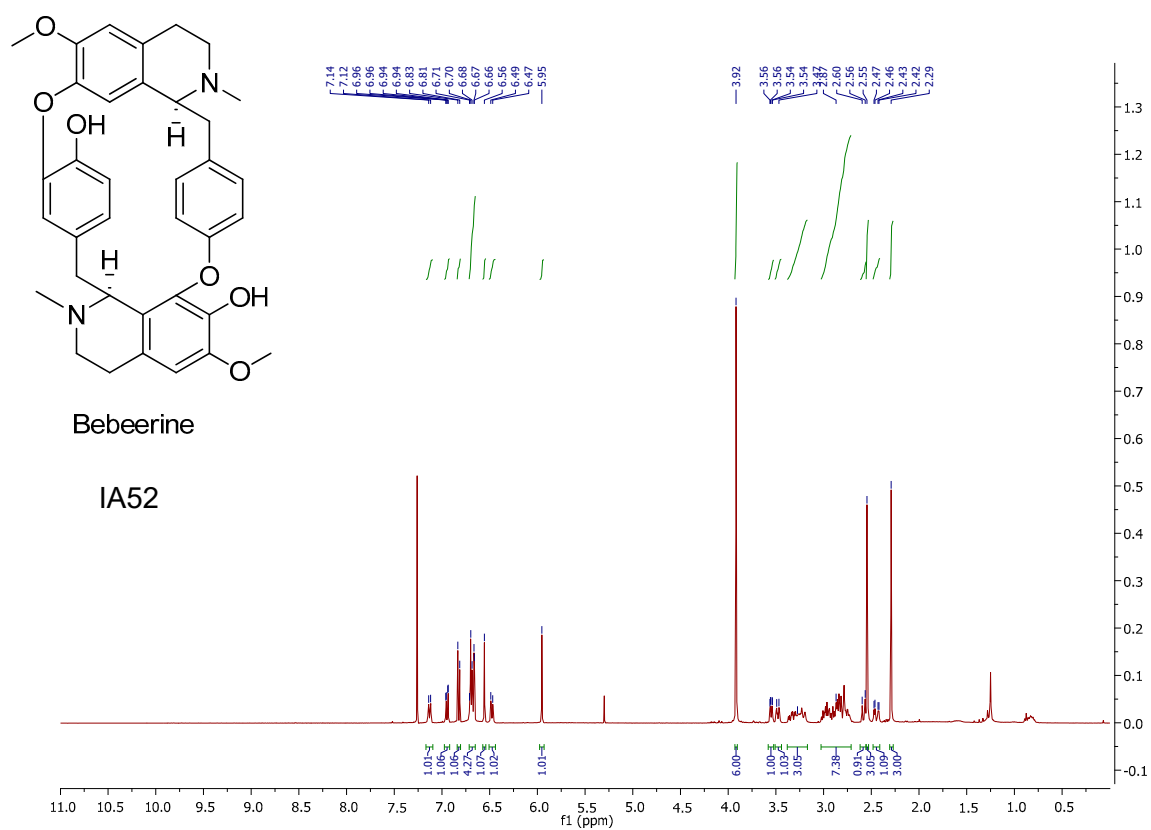

**Supplementary Figure S4.** <sup>1</sup>H NMR spectrum of IA52 (bebeerine) recorded in CDCl<sub>3</sub> at 300 MHz.

Supplement: Supplementary file 1 [file molecules-27-04133-s001.zip › Figure S4 RMN-bebeerine.pdf]

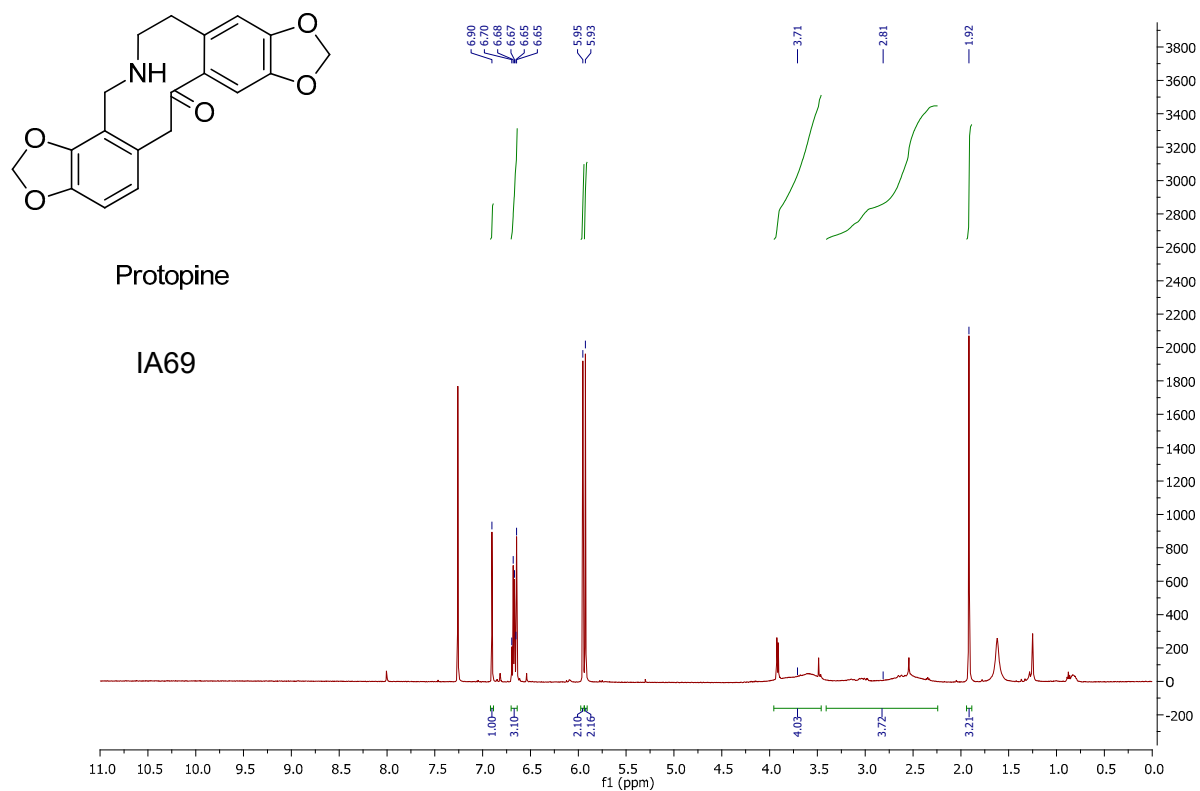

**Supplementary Figure S5.**  $^1\text{H}$  NMR spectrum of IA69 (protopine) recorded in  $\text{CDCl}_3$  at 300 MHz.

Supplement: Supplementary file 1 [file molecules-27-04133-s001.zip › Figure S5-RMN protopine.pdf]
